# Ecotone Might Provide Key Refugium for Sky Island Mammals in the Southern Appalachian Mountains

**DOI:** 10.1002/ece3.72374

**Published:** 2025-10-28

**Authors:** Jenifer A. Mallinoff, Radmila Petric, Corinne A. Diggins, Elizabeth M. Kierepka, Brian S. Arbogast, Andrew Jenkins, Marketa Zimova

**Affiliations:** ^1^ Department of Biology Appalachian State University Boone North Carolina USA; ^2^ The Nature Conservancy Asheville North Carolina USA; ^3^ Institute for the Environment University of North Carolina Chapel Hill Chapel Hill North Carolina USA; ^4^ Science Applications, Southwest Region U.S. Fish and Wildlife Service Albuquerque New Mexico USA; ^5^ North Carolina Museum of Natural Sciences Department of Forestry and Environmental Resources, North Carolina State University Raleigh North Carolina USA; ^6^ Department of Biology and Marine Biology University of North Carolina Wilmington North Carolina USA; ^7^ Biological Sciences Department Ohio University Athens Ohio USA

**Keywords:** American red squirrel, Appalachian Mountains, biodiversity hotspot, Carolina northern flying squirrel, cloudland deer mouse, Fraser fir, red spruce, southern red‐backed vole

## Abstract

Sky islands, ecosystems found on geographically isolated mountain peaks, are among the most biodiverse ecosystems in the world but face a disproportionately high threat from climate change. High‐elevation, montane ecosystems, which are already at their upper altitudinal limits, are predicted to severely contract in response to climate change. The identification and conservation of refugia is an increasingly important approach for protecting biodiversity associated with imperiled ecosystems. We explored the spruce‐fir–northern hardwood ecotone as a possible refugium for mammals in the Southern Appalachian red spruce ( *Picea rubens* )‐Fraser fir ( *Abies fraseri* ) sky islands. We conducted livetrapping, camera trapping, and ultrasonic acoustic surveys to characterize mammal diversity across the spruce‐fir–northern hardwood forest gradient on Grandfather Mountain and Roan Mountain Highlands in western North Carolina, USA. We detected four out of the five spruce‐fir‐associated small mammal species in both spruce‐fir and ecotone habitats. Mammal species richness, alpha diversity, and bat activity tended to be higher in the ecotone than in the other forest types on both mountains. Next, the abundance of small mammals associated with spruce‐fir was higher in the spruce‐fir and ecotone forests for one of the three species we were able to estimate. Together, our results suggest that the spruce‐fir–northern hardwood ecotone might serve as refugium for mammal species that are associated with spruce‐fir sky islands in the Southern Appalachian Mountains and mammalian conservation efforts in this biodiversity hotspot should consider focusing on the ecotone in addition to the adjacent spruce‐fir ecosystem.

## Graphical abstract

Sky islands are among the most biodiverse ecosystems in the world but face a disproportionately high threat from climate change. We examined the spruce‐fir–northern hardwood ecotone as a possible refugium for mammals in the Southern Appalachian red spruce ( *Picea rubens* )–*Fraser* fir ( *Abies fraseri* ) sky islands. We detected spruce‐fir‐associated small mammal species in both spruce‐fir and ecotone habitats, wherein mammal species richness, alpha diversity, and bat activity tended to be higher in the ecotone than in the other forest types on both mountains. Together, our results suggest that the spruce‐fir–northern hardwood ecotone may serve as a key refugium for mammal species that are associated with spruce‐fir sky islands in the Southern Appalachian Mountains, and mammalian conservation efforts in this biodiversity hotspot should consider focusing on this ecotone in addition to the adjacent spruce‐fir ecosystem.

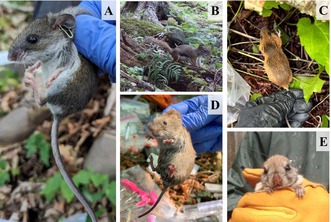

## 1 Introduction

Anthropogenic climate change is a major threat to global biodiversity (Dawson et al. 2011; Mantyka‐Pringle et al. 2015; Pacifici et al. 2015). Sky islands, ecosystems found on geographically isolated mountain peaks, face a disproportionately high threat of extirpation due to anthropogenic climate change (Love et al. 2023). These ecosystems exist at their elevational limits and are expected to rapidly contract with climatic warming and drying (Delcourt and Delcourt 1987; Noss et al. 1995). Yet, sky islands are among the most biodiverse ecosystems in the world (Love et al. 2023), and targeted conservation measures are necessary to protect the lineages of rare, endemic, and threatened species that occupy them.

The identification and protection of habitats that may allow species to persist in the future, including climate‐change refugia, has increasingly been proposed as a conservation strategy to support the persistence of biodiversity under climate change (Keppel et al. 2012, 2024; Morelli et al. 2016, 2020). In broader terms, refugia are habitats that may facilitate the persistence of species during large‐scale, long‐term climatic change (Keppel et al. 2012, 2015). Ecotones, the transitional zones between two ecosystems (Kupfer and Cairns 1996), are rarely examined as potential refugia for species associated with the adjacent threatened ecosystems (Killeen and Solórzano 2008). Yet, ecotones often harbor high biodiversity due to their diverse habitat features and increased structural complexity (Risser 1995; Chapman et al. 1996; Killeen and Solórzano 2008). Because they contain elements from both neighboring ecosystems, ecotones provide habitat and resources for species linked to one environment but not the other, ultimately often supporting greater biodiversity than either ecosystem alone (Kark 2013). Better understanding of the role ecotones may play in the persistence of species whose habitats are disappearing, including those associated with sky islands, is needed to inform biodiversity conservation under climate change.

Southern Appalachian spruce‐fir sky islands are biodiverse, highly threatened ecosystems (Stein et al. 2000; Burns et al. 2003; Jenkins et al. 2015). They are a relic of the boreal forests that were dominant across much of the Southeastern United States at the end of the Last Glacial Maximum (10,000–12,000 years before present) and occur on high‐elevation peaks and ridgelines in the region (Delcourt and Delcourt 1987). Exploitative logging and subsequent wildfires at the turn of the 20th century, air pollution, and the invasive balsam woolly adelgid (*Adelges piceae*) have critically reduced the extent of the spruce‐fir sky islands (Korstian 1937; Schafale and Weakley 1990; Noss et al. 1995; Delcourt and Delcourt 1987, 1998; Koo et al. 2015). In the Southern Appalachians, spruce‐fir sky islands are predicted to continue contracting upslope or disappear altogether under most climate‐change scenarios, being replaced by the spruce‐fir–northern hardwood ecotone and the northern hardwood forests that dominate lower elevations (Noss et al. 1995; Delcourt and Delcourt 1998; Iverson et al. 2008; Koo et al. 2015).

**FIGURE 1:**
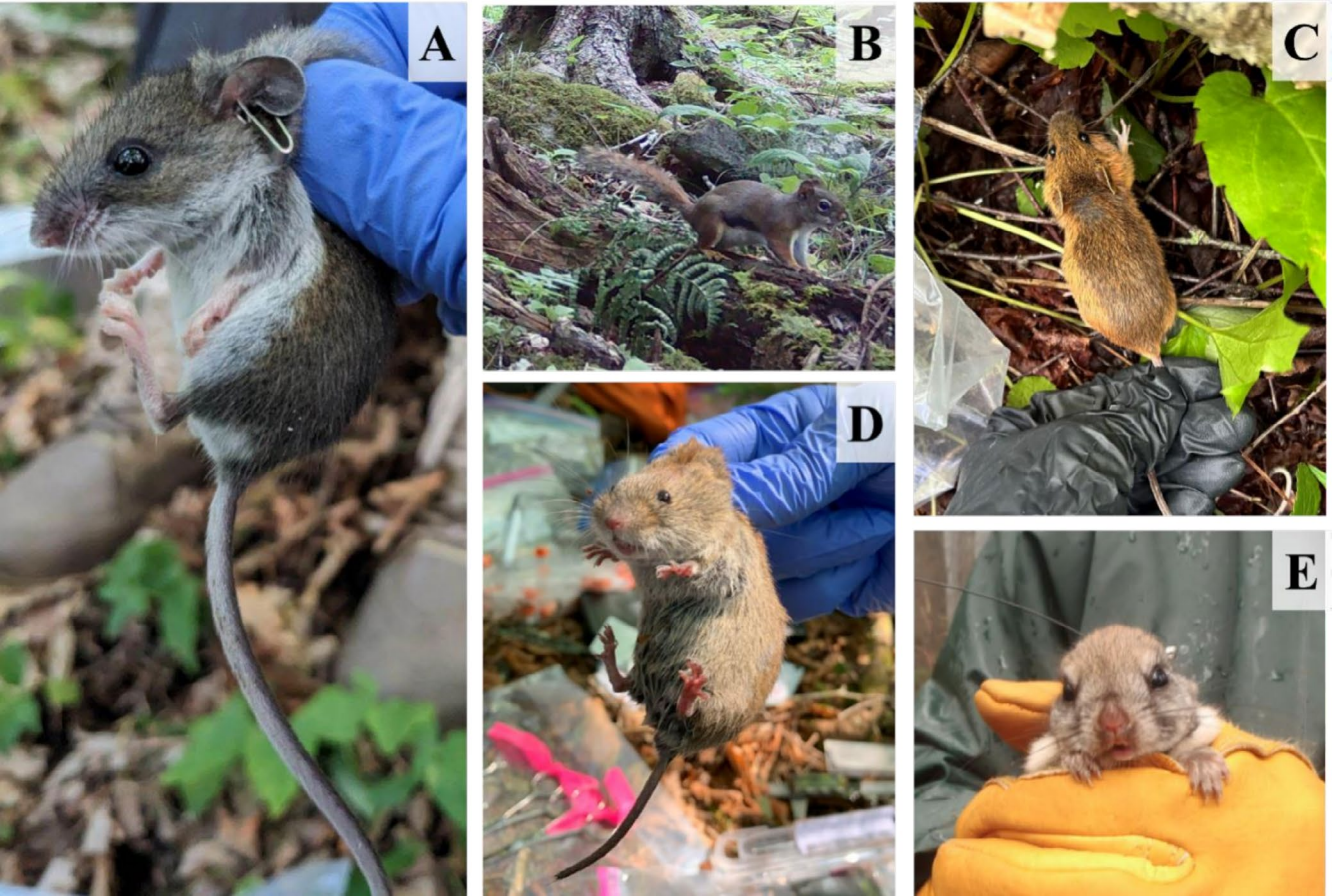
Small mammal species associated with high‐elevation spruce‐fir forests in the Southern Appalachian Mountains. (A) Cloudland deer mouse (*Peromyscus maniculatus nubiterrae*); photo credit: J. Mallinoff, (B) American red squirrel ( *Tamiasciurus hudsonicus* ; photo credit: J. Mallinoff), (C) woodland jumping mouse ( *Napaeozapus insignis* ; photo credit: E. Claire Watersmith), (D) southern red‐backed vole ( *Clethrionomys gapperi* ; photo credit: J. Mallinoff), and (E) the federally endangered Carolina northern flying squirrel ( *Glaucomys sabrinus coloratus* ; photo credit: Corinne Diggins/USGS 2014).

Southern Appalachian spruce‐fir sky islands harbor a unique assemblage of endemic and locally rare, as well as generalist, mammal species, including numerous bat species (Steele and Powell 1999; Laerm et al. 2000; Browne and Ferree 2007; Campbell et al. 2010; Ford et al. 2014; Graeter et al. 2015; Diggins et al. 2017; Diggins and Ford 2022). Among the specialists associated with high‐elevation spruce‐fir forests are the southern red‐backed vole ( *Clethrionomys gapperi* ), cloudland deer mouse (*Peromyscus maniculatus nubiterrae*), American red squirrel ( *Tamiasciurus hudsonicus* ), woodland jumping mouse ( *Napaeozapus insignis* ), and the federally endangered Carolina northern flying squirrel ( *Glaucomys sabrinus coloratus* ; Figure 1), all of which play key ecological roles, including seed and fungal spore dispersal, serving as prey species of raptors and carnivores, and, in the case of American red squirrels, predators of bird eggs and nestlings (Steele and Powell 1999; Arbogast et al. 2005; Browne and Ferree 2007; Campbell et al. 2010; Ford et al. 2014; Diggins et al. 2016, 2017; Stephens and Rowe 2020; Morelli et al. 2025). These species vary in their dependence on spruce‐fir forests, ranging from the Carolina northern flying squirrel that is highly dependent on this habitat (Ford et al. 2014; Diggins et al. 2017) to the American red squirrel and woodland jumping mouse, which in this region also occur in lower‐elevation forests (Browne and Ferree 2007; Linzey 2016; Morelli et al. 2025). However, many spruce‐fir specialists are likely confined to the high‐elevation sky islands, and their limited dispersal capacity prevents them from tracking their preferred habitat across fragmented landscapes, making them especially vulnerable to climate change. Indeed, mammals are among the taxa most at risk from climate change in the Southern Appalachians (Zhu et al. 2022). Yet our understanding of their current distribution and community composition in these forests remains limited, especially compared with other imperiled taxa (Adams and Hammond 1991; Appelget 1993; Steele and Powell 1999; Campbell et al. 2010).

Here, we used multiple field survey methods to characterize mammal diversity across the spruce‐fir–northern hardwood forest gradient and to assess whether sky‐island specialists currently occupy the intervening spruce‐fir–northern hardwood ecotone. Despite occurring at lower elevations, this ecotone likely provides the resources (e.g., spruce‐fir cover, food availability) and microclimates important for spruce‐fir‐associated mammals, potentially serving as a key refugium. Furthermore, in many regions, the ecotone is expected to persist much longer under climate change, with upward expansion already having been recorded in some areas (Mayfield III and Hicks Jr 2009; Rollins et al. 2010; Foster and D'Amato 2015), though this response may not be uniform across the landscape. Surveying across the spruce‐fir–northern hardwood gradient on two mountains in the Southern Appalachian Mountains (Grandfather Mountain, Roan Mountain Highlands), we quantified (1) mammal species richness and alpha diversity, (2) small mammal species abundance, and (3) activity of flying squirrels and bats. We also examined whether spruce‐fir‐associated species occur in the ecotone. Together, we used our findings to evaluate the potential role of the spruce‐fir–northern hardwood ecotone as a refugium for mammal biodiversity associated with Southern Appalachian spruce‐fir sky islands.

## 2 Methods

### 2.1 Study Area

This study was conducted in the Southern Appalachian Mountains, in western North Carolina, USA. We surveyed mammal communities on two mountains: Grandfather Mountain (36°6′ N and 81°48′ W; 1812 m above sea level [asl]), and Roan Mountain Highlands (36°6′ N and 82°7′ W; 1916 m asl). On each mountain, we conducted surveys at three sites along an elevational gradient, each located within one of the following forest types: spruce‐fir, spruce‐fir–northern hardwood ecotone, and northern hardwood. We surveyed six sites in total.

The peaks of Grandfather and Roan Mountain Highlands (approximately 1600–2000 m asl) consist of red spruce ( *Picea rubens* ) and Fraser fir ( *Abies fraseri* ) sky islands with some hardwood tree species present, including yellow birch ( *Betula alleghaniensis* ) and American mountain ash ( *Sorbus americana* ). These spruce‐fir forests experience cooler temperatures, higher precipitation, and more frequent cloud immersion than lower‐elevation forests (Smith 2006; Koo et al. 2015). Below approximately 1600 m asl, the forest transitions into a spruce‐fir–northern hardwood ecotone with the appearance of more northern hardwood tree species (Simon et al. 2005; Smith 2006; Schafale 2012). While red spruce remains a common component of the ecotone, northern hardwood species yellow birch and sugar maple ( *Acer saccharum* ) become more dominant in the canopy, and Fraser firs occur rarely. Other northern hardwood species also appear, including American beech ( *Fagus grandifolia* ) on Roan Mountain Highlands and black cherry ( *Prunus serotina* ), striped maple ( *Acer pensylvanicum* ), and white ash ( *Fraxinus americana* ) on Grandfather Mountain. At lower elevations (approximately 1200–1600 m asl; Smith 2006), the ecotone transitions into northern hardwood forest with the absence of red spruce and Fraser fir and the addition of more hardwood tree species such as yellow buckeye ( *Aesculus flava* ).

### 2.2 Mammal Surveys

**FIGURE 2:**
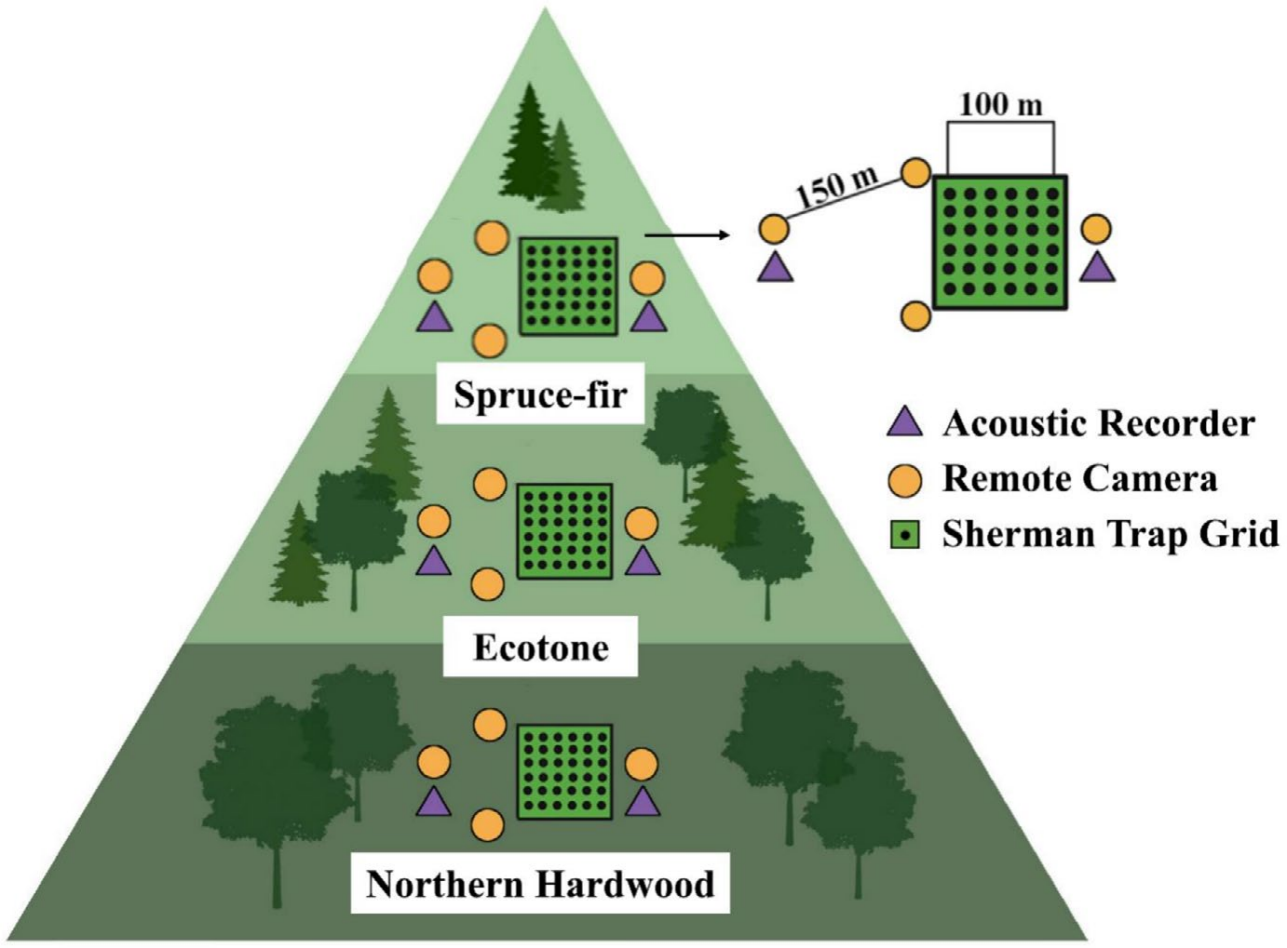
Field survey schematic for surveying mammals across the spruce‐fir–northern hardwood gradient in montane sky islands in the Southern Appalachian Mountains of western North Carolina, USA, in summer 2023. Each mountain had three survey sites, one located within each forest type. Each survey site included one livetrapping grid, four remote wildlife camera traps, and two ultrasonic acoustic recorders. The grid on the right shows the survey site design that was repeated in each forest type. The numbers indicate distances in meters. We surveyed a total of six sites in this study.

We surveyed mammals along an elevational gradient at each mountain, from 1324 to 1635 m asl on Grandfather Mountain and 1547 to 1872 m asl in the Roan Mountain Highlands (Table A1 in Appendix A). The peaks of Grandfather and Roan Mountains were approximately 27 km straight‐line distance from one another. We used three field survey techniques to document volant and non‐volant mammal species within each forest type on each mountain: live traps, remote camera traps, and ultrasonic acoustic recorders (Figure 2). All surveys occurred in June and July 2023 on the southern aspect of both mountains (Table A1 in Appendix A). We surveyed a total of six sites (three forest types on each mountain) that were spaced by 0.6–1.9 km. Our protocol conformed to the guidelines outlined by the American Society of Mammalogists (Sikes et al. 2016) and was approved by the Appalachian State University Institutional Animal Care and Use Committee (permit #22‐12) with permissions from relevant management authorities.

#### 2.2.1 Livetrapping

We live‐trapped non‐volant small mammal species using Sherman traps (3″ × 3.5″ × 9″; H.B. Sherman Traps, Tallahassee, FL, USA). Each survey site consisted of 36 traps arranged in a 6 × 6 grid with 20 m spacing between traps (Figure 2). We set traps at sunset and checked and closed them at sunrise. Each trapping session consisted of four consecutive trap nights for a total of 144 trap nights per each 100 m^2^ survey site. We baited traps with sunflower seeds, oats, and mealworms, and placed a cotton ball into each trap to help animals thermoregulate during colder nights. We placed traps at grid intersections in proximity to woody debris, trees, or rocks (Converse et al. 2006).

For each captured rodent, we measured weight, hind foot length, body length, and tail length for species identification (Stephens et al. 2014; Berl et al. 2017), and determined sex, age class (adult or juvenile), and external reproductive status (Steele and Powell 1999; Polyakov et al. 2021). We uniquely marked each adult rodent in the right ear with an ear tag (Style 1005‐1L1, National Band & Tag Company, Newport, KY, USA; McCain 2004) and used surgical scissors to take ear tissue samples from *Peromyscus* for molecular species assignment (see “Molecular species assignment and discriminant function analysis” section below). Shrews were weighed, sexed, and identified to species. We used a permanent ink pen to mark juvenile rodents and all shrews with a unique symbol on the stomach or tail. All animals were released at their capture location.

#### 2.2.2 Molecular Species Assignment and Discriminant Function Analysis

Morphology‐based identification is often unreliable for the two *Peromyscus* species in the region, the cloudland deer mouse (*P. maniculatus nubiterrae*) and the white‐footed mouse ( *P. leucopus* ) (Choate 1973; Stephens et al. 2014; Berl et al. 2017). We extracted DNA from the collected *Peromyscus* ear tissue samples via Qiagen DNeasy blood and tissue kits (Qiagen, Germantown, MD, USA). A random subset of the DNA extracts (*n* = 48) was analyzed at the North Carolina Museum of Natural Sciences for identification to species using the cytochrome b mitochondrial gene (primers: MTCB‐F and R; Naidu et al. 2012).

For DNA sequencing, polymerase chain reactions (PCR) occurred in 10 μL volumes consisting of 5–10 ng of extracted DNA, 10 nM of forward and reverse primer, 2.0 mM of MgCl_2_, 5.0 μL of 1:5 mix of Takara Taq Polymerase and Promega GoTaq MasterMix, and 2.9 μL nuclease‐free water. PCRs began with initiation at 95°C for 2 min, then 35 cycles of denaturing at 95°C for 15 s, annealing at 52°C for 30 s, and extension at 72°C for 1 min with a final 10‐min extension at 72°C. We sequenced the forward primer for cytochrome b as it yields sufficient power to discern *Peromyscus* species (~600 bp of cytochrome b). Sequencing reactions initiated at 96°C for 3 min, then underwent 30 cycles at 96°C for 10 s, 50°C for 5 s, and 60°C for 2.5 min. We used the ethanol precipitation method to clean sequencing reactions (Latch and Rhodes 2005) and then sequenced them on a 3500 Genetic Analyzer (Applied Biosystems, Foster City, CA, USA). We aligned all sequences in Geneious Prime (Kearse et al. 2012) and uploaded them to GenBank (Accession Numbers PV487868–PV487919).

Finally, to identify the remaining live‐trapped *Peromyscus* individuals to species, we used a linear discriminant function analysis model in the “mda” 0.5‐4 package (Hastie et al. 2023) in R 4.3.2 (R Core Team 2023). The model function was built using four body measurements (weight, hind foot length, body length, and tail length). Analysis of variance tests confirmed that each body measurement differed significantly between the two species (*p* < 0.01). The model correctly assigned species identity for 93.9% of the 48 individuals previously identified by DNA analysis, and we used it to predict the species of the remaining captured individuals (*n* = 100).

#### 2.2.3 Remote Camera Trapping

We used remote camera traps to detect small to large non‐volant mammal species. We installed four remote cameras (Spec Ops Elite HP5, Browning, Morgan, UT, USA) at each survey site within each forest type so that cameras were at a minimum of 150–200 m apart (Figure 2). We mounted each camera at 30–50 cm on the bole of a tree, following standard methodology to survey for small to large mammals (Evans and Mortelliti 2022; Rooney et al. 2025) near animal trails, rock outcrops, logs, and other desirable habitat features to increase the probability of detection (Trolle et al. 2008; Gebert et al. 2019; Hofmeester et al. 2021). We set cameras to take five‐image photo bursts every 5 s when motion was detected. We considered all images taken of the same species at the same camera within an hour as one detection (Hegerl et al. 2017; Gebert et al. 2019). We set the cameras at each site for 31 days, for a total of 372 trap nights per mountain. We uploaded, sorted, and manually identified species in all camera images in Wildlife Insights (https://www.wildlifeinsights.org/; Ahumada et al. 2020).

#### 2.2.4 Ultrasonic Acoustic Monitoring

We used ultrasonic acoustic detectors (SM4, Wildlife Acoustics Inc., Maynard, MA, USA) to record vocalizations and quantify bat activity and species richness, and the activity of American flying squirrels (Carolina northern flying squirrel and southern flying squirrel; Diggins et al. 2016, 2020) across the forest types. We installed two acoustic detectors at each trap grid in the same location as the camera traps (Figure 2). To detect bats and American flying squirrels while minimizing the detection of nontarget species, we set detectors to record sounds with a minimum frequency of 16 kHz and minimum length of 1.5 ms from 30 min before sunset to 30 min after sunrise for 10 days (Gilley et al. 2019; U.S. Fish and Wildlife Service 2024). We attached detectors to tree trunks 1.5 m off the ground, facing the direction with the least amount of clutter to improve recording quality (Diggins et al. 2016, 2020). We conducted acoustic recording for a total of 10 consecutive nights per survey site.

We used SonoBat software (SonoBat 4.4.5, DND Design, Arcata, CA, USA) to sort and identify flying squirrel calls. We confirmed all species identifications using two observers. A total of 845 calls were identified as American flying squirrels. We were able to identify four flying squirrel call types: chirps, trills, upsweeps, and crows (Gilley et al. 2019). We identified bat passes to species using Kaleidoscope Pro Analysis Software (Kaleidoscope 4.4, Wildlife Acoustics Inc., Maynard, MA, USA). Each bat pass had to match a species identification accuracy of at least 60% or the pass was classified as a “no ID” (Li and Kalcounis‐Rueppell 2018; Schimpp et al. 2018; Parker et al. 2019). We estimated bat species activity at each site as the mean number of bat passes recorded per night over the 10‐night survey period. We identified a total of 6436 bat passes to species.

### 2.3 Vegetation Surveys

We conducted vegetation surveys at every survey site to characterize each forest type. We established a circular 400 m^2^ plot centered at each live‐trap grid. In every plot, all live trees (≥ 1.4 m height) were identified to species and measured at diameter at breast height. In addition, we counted the number of tree snags. Within each 400 m^2^ plot, we established a 40 m^2^ subplot where all live saplings (< 1.4 m height) were identified to species and dead saplings counted (Kalies et al. 2012). We used the line interception method (Canfield 1941) along four perpendicular transects within the subplot to identify shrubs and herbaceous plant species. We calculated plant species richness as the total number of species detected at each survey site, including tree, shrub, and herbaceous species. We also calculated the percentage of trees that consisted of Fraser fir, red spruce, and northern hardwood species. Finally, we measured slope and aspect at the center of each plot.

### 2.4 Statistical Analysis

#### 2.4.1 Mammal Species Richness and Diversity

We estimated mammal species richness and alpha diversity at each survey site and mountain using our field data. Because we surveyed only one site per forest type per mountain, we did not perform formal statistical comparisons of these metrics or of the other variables described below (small mammal abundance, flying squirrel activity, and bat activity). Species richness within each forest type on each mountain was calculated by counting the number of species detected at each site using all survey data (livetrapping, remote camera trapping, and acoustic recording data). We calculated richness for all mammal species combined and richness for volant and non‐volant species separately, to ensure that species richness of either group was not driving overall trends. Next, we used the camera‐trapping data to estimate multiple alpha diversity indices using the Community Ecology Package “vegan” 2.6‐4 (Oksanen et al. 2022) in R 4.3.2 (R Core Team 2023). We limited the alpha diversity estimates to the camera‐trapping data (i.e., non‐volant species) because this method allowed us to account for the relative abundance of each species. We computed three alpha diversity indices at each site: Shannon diversity (*H′* = −Σ*p*_*i*_ln(*p*_*i*_)), Simpson's diversity (*D* = 1 − Σ*p*_*i*_^2^), and Pielou's evenness (*J'* = *H′*/*H*_max_), where *p*_*i*_ = the relative abundance of species *i* and *H*_max_ = maximum possible Shannon index (the natural log of species richness) (Shannon and Weaver 1949; Simpson 1949; Pielou 1966). We chose these indices for a comprehensive view of alpha diversity, where Shannon diversity stresses the presence of rarer species, Simpson's diversity emphasizes common species, and Pielou's evenness measures how evenly the number of individuals is distributed among the species.

#### 2.4.2 Small Mammal Abundance

We used the Schnabel method to estimate small mammal species abundance at each site. The Schnabel method follows the equation *N* = Σ(C*M)/Σ*R*, where *C* = number of total captures on each trap occasion, *M* = number of marked individuals on each occasion, and *R* = number of recaptures on each occasion, which makes the same assumptions as the Lincoln–Petersen method while also explicitly accounting for multiple recapture events (Schnabel 1938; Krebs 1999). We calculated 95% confidence intervals using the Poisson distribution, as we recaptured fewer than 50 individuals for every capture event (Krebs 1999). We also computed abundance using the Lincoln–Petersen method, which produced similar results (Table A2 in Appendix A).

#### 2.4.3 Flying Squirrel and Bat Activity

We calculated flying squirrel activity as the total number of calls recorded at each survey site. We calculated bat activity as the mean number of recorded passes per night at each survey site.

## 3 Results

### 3.1 Mammal Species Richness and Diversity

**FIGURE 3:**
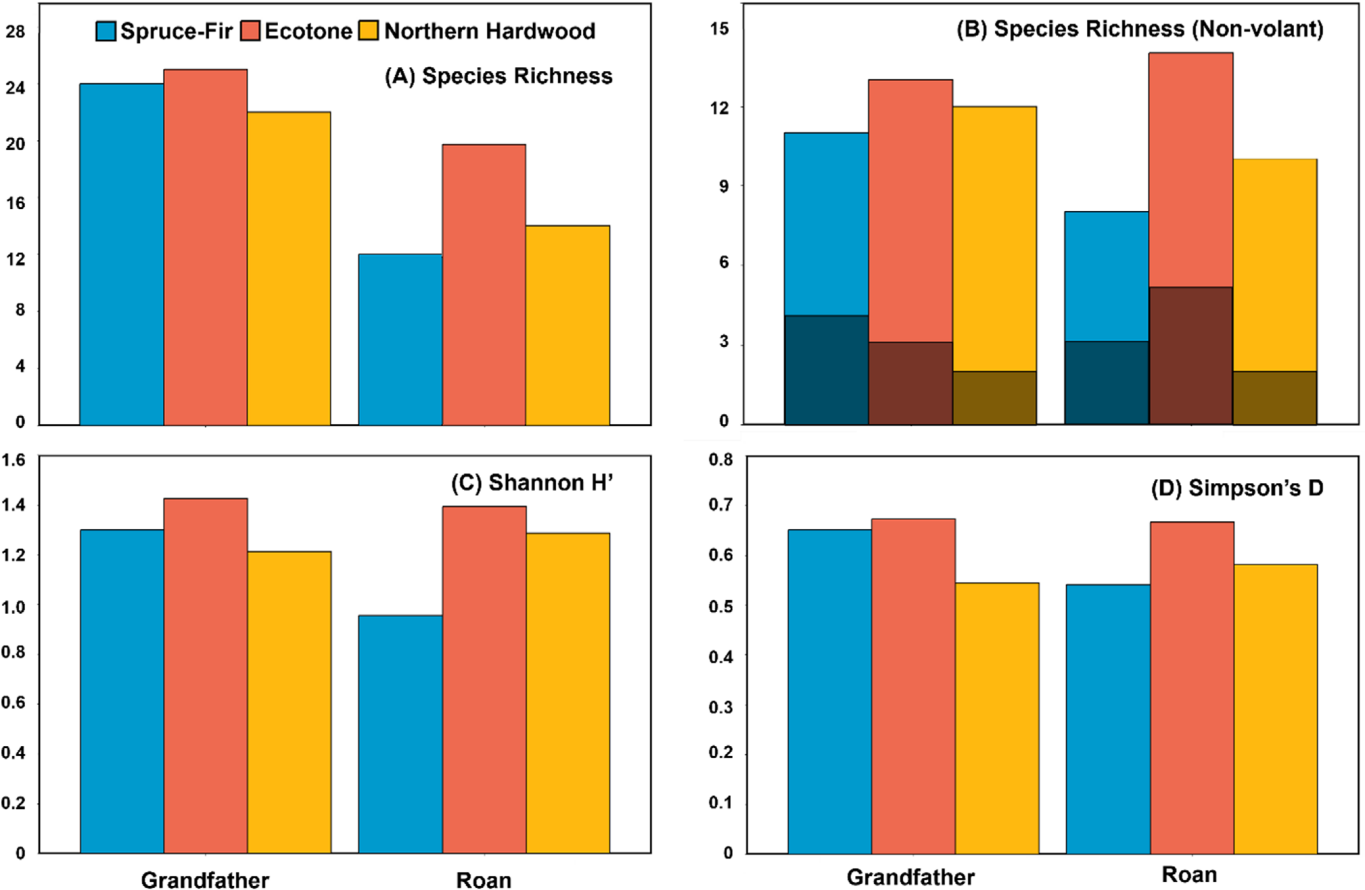
Mammal species richness and alpha diversity indices for the three forest types on Grandfather Mountain and Roan Mountain Highlands in the Southern Appalachian Mountains of North Carolina, USA. Species richness was calculated separately for all mammals (A) and for non‐volant mammals (B) using the data from all survey methods (livetrapping, camera trapping, and acoustic recording) conducted in summer 2023. The shaded regions in (B) depict spruce‐fir‐associated species (Carolina northern flying squirrel *Glaucomys sabrinus coloratus* , southern red‐backed vole *Clethrionomys gapperi* , cloudland deer mouse *Peromyscus maniculatus nubiterrae*, woodland jumping mouse *Napaeozapus insignis* , and American red squirrel *Tamiasciurus hudsonicus* ). (C) Shannon Diversity H′ and (D) Simpson's Diversity D were estimated using camera‐trapping data only. For similar results based on Pielou's evenness *J*' see the Figure A1 in Appendix A.

**FIGURE A1:**
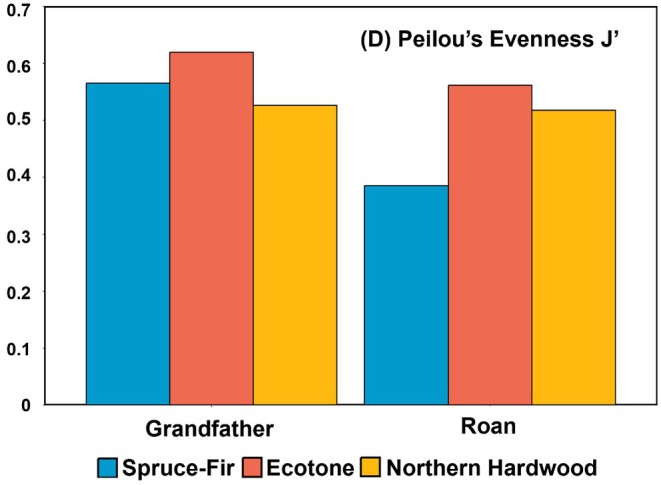
Peilou's Evenness J' of non‐volant mammals across three forest types surveyed at Grandfather Mountain and Roan Mountain Highlands in western North Carolina, USA summer 2023. Evenness is estimated with camera trapping data only.

Using all three survey methods (livetrapping, remote camera trapping, and ultrasonic acoustic recording), we detected a total of 33 mammal species across the two mountains (for detailed information, see Table A4 in Appendix A). Within the surveyed sites, species richness and all alpha diversity indices (Shannon index *H*', Simpson's index *D*, and Pielou's evenness *J*') tended to be higher in the ecotone than in the other forest types on both mountains (Figure 3; Figure A1 in Appendix A). Similarly, species richness was higher in the ecotone on both mountains, even when bats (13 species) and non‐volant mammals (20 species) were analyzed separately (Figure 3B). We detected five spruce‐fir‐associated species (Carolina northern flying squirrel, southern red‐backed vole, woodland jumping mouse, cloudland deer mouse, and American red squirrel) in both spruce‐fir and ecotone forests but only two (American red squirrel and cloudland deer mouse) in the northern hardwood forests on both spruce‐fir sky islands (Figure 3B; Table A4 in Appendix A).

### 3.2 Small Mammal Abundance

**FIGURE 4:**
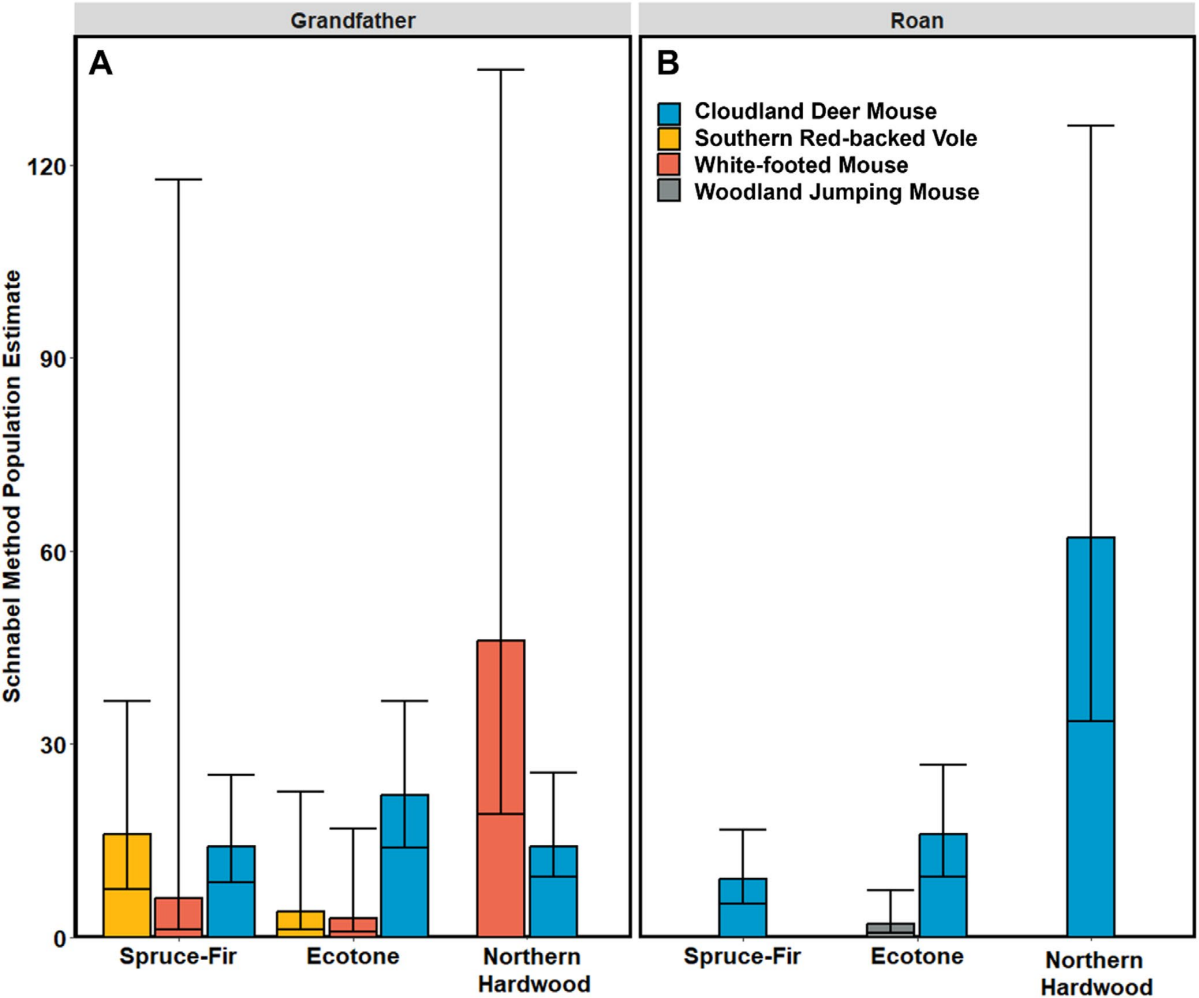
Small mammal species abundance from montane sky islands in the Southern Appalachian Mountains of western North Carolina, USA, in summer 2023. Abundance estimates are shown with 95% confidence intervals using the Schnabel method of population estimation. Abundances for the two study sites for small mammals (cloudland deer mouse *Peromyscus maniculatus nubiterrae*, southern red‐backed vole *Clethrionomys gapperi* , white‐footed mouse *Peromyscus leucopus* , woodland jumping mouse *Napaeozapus insignis* ) are shown for each mountain: Grandfather Mountain (A) and Roan Mountain Highlands (B). Raw capture data for each species and abundance estimates calculated using the Lincoln–Petersen method of abundance estimation are available in the Tables A2 and A3 in Appendix A.

We were able to quantify abundance for three spruce‐fir‐associated species (southern red‐backed vole, cloudland deer mouse, woodland jumping mouse) and one low‐elevation species (white‐footed mouse). On Grandfather Mountain, southern red‐backed voles had the highest estimated abundance in the spruce‐fir site, followed by the ecotone site, and were absent from the northern hardwood forest (Figure 4A). Next, the abundance of cloudland deer mice on Grandfather Mountain was similar across all three forest types (Figure 4A), while the abundance of white‐footed mice was highest in the northern hardwood forest (Figure 4A). On Roan Mountain Highlands, the abundance of cloudland deer mice was highest in the northern hardwood forest and much lower at the other two vegetation types (Figure 4B). Woodland jumping mice were present only in the ecotone site on Roan Mountain Highlands. We were not able to estimate the abundance of white‐footed mice and southern red‐backed voles on Roan Mountain Highlands due to low capture numbers.

### 3.3 Flying Squirrel and Bat Activity

**FIGURE 5:**
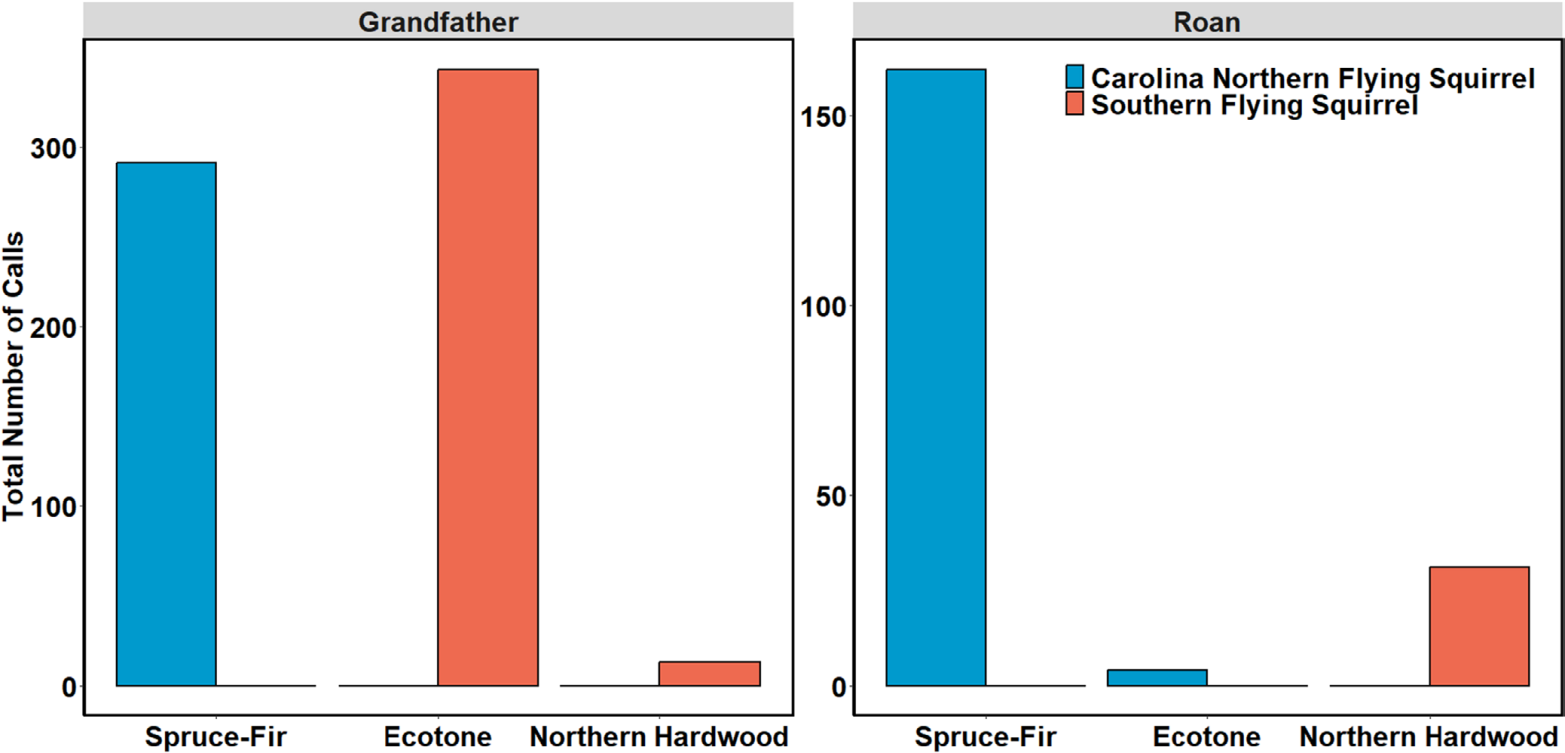
Total number of Carolina northern flying squirrel ( *Glaucomys sabrinus coloratus* ) and southern flying squirrel ( *Glaucomys volans* ) calls detected with ultrasonic acoustic detectors at Grandfather Mountain (A) and Roan Mountain Highlands (B) in the Southern Appalachian Mountains of western North Carolina, USA in summer 2023. Raw counts are available in Table A5 in Appendix A.

American flying squirrel species were present on both mountains, although the number of recorded calls was three times higher on Grandfather Mountain than on Roan Mountain Highlands. We identified 845 American flying squirrel calls (Figure 5; Table A5 in Appendix A). We detected all but four of the 457 Carolina northern flying squirrel calls in the spruce‐fir forest on both sky islands (Table A5 in Appendix A). We detected southern flying squirrels in the ecotone and northern hardwoods on Grandfather Mountain and only in the northern hardwoods on Roan Mountain Highlands (*n* = 387, Table A5 in Appendix A).

**FIGURE 6:**
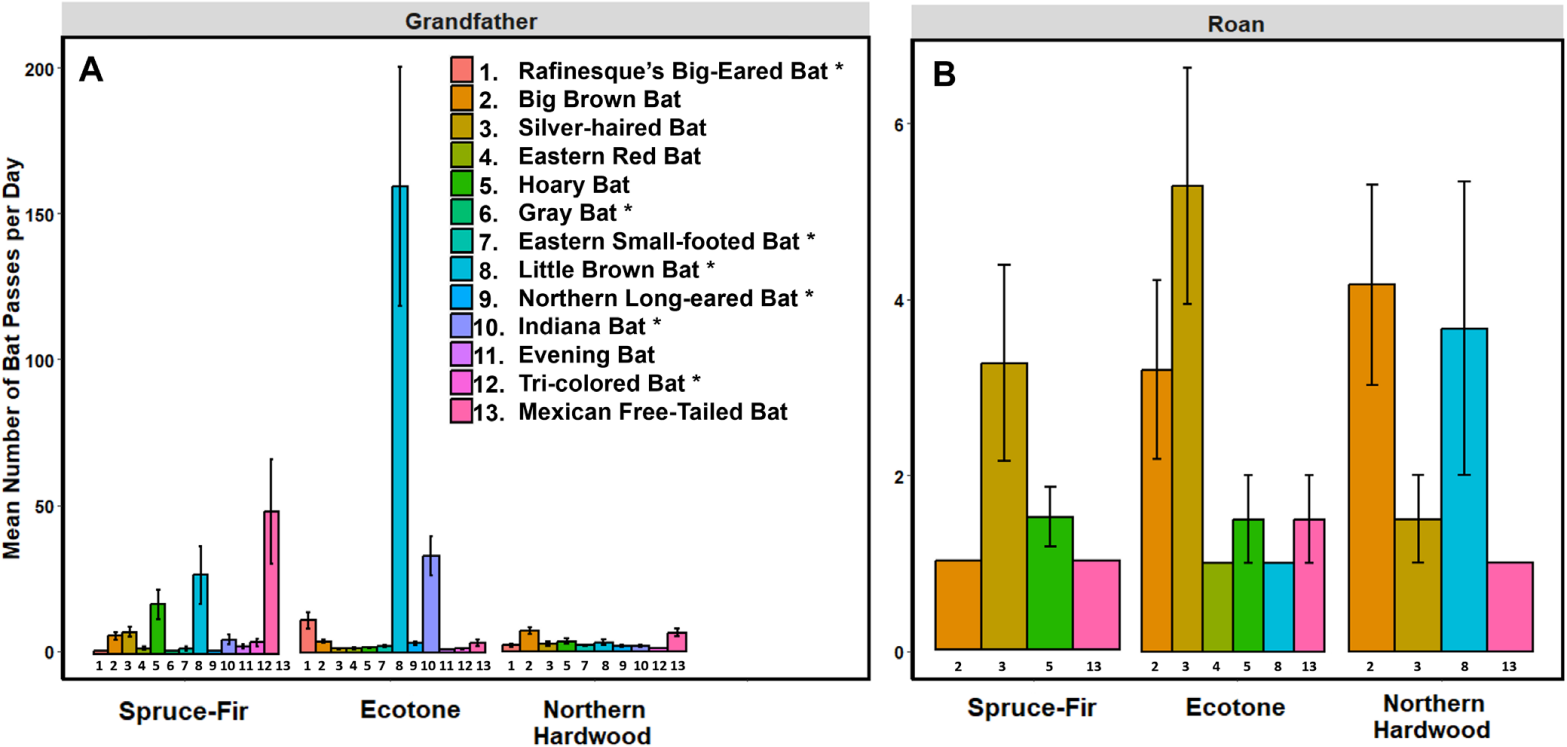
Mean bat activity (mean number of passes per night) in montane sky islands in the Southern Appalachian Mountains of western North Carolina in summer 2023. Data is presented with standard error bars. Activity is shown for red spruce ( *Picea rubens* )‐Fraser fir ( *Abies fraseri* ) forest, ecotonal forest, and northern hardwood forest at Grandfather Mountain (A) and Roan Mountain Highlands (B). Species of special concern include Rafinesque's big‐eared bat ( *Corynorhinus rafinesquii* ) and eastern small‐footed bat (*Myotis leibii*); North Carolina state endangered tricolored bat (*Perimyotis subflavus*) and little brown bat (*Myotis lucifugus*); federally endangered gray bat ( *Myotis grisescens* ), Indiana bat ( *Myotis sodalis* ), northern long‐eared bat ( *Myotis septentrionalis* ) (North Carolina Wildlife Resources Commission 2021). Asterisk indicates species of conservation concern, North Carolina state endangered and federally endangered species.

Bat activity tended to be higher in the ecotones than in the other forest types (Figure 6). We detected 12 species in the ecotone site on Grandfather Mountain and six in the ecotone site on Roan Mountain Highlands (Figure 6). We detected seven species of special concern, North Carolina state endangered, and federally endangered (Rafinesque's big‐eared bat *Corynorhinus rafinesquii* , gray bat *Myotis grisescens* , eastern small‐footed bat *Myotis leibii*, little brown bat *Myotis lucifugus*, northern long‐eared bat *Myotis septentrionalis*, Indiana bat *Myotis sodalis* , tricolored bat *Perimyotus subflavus*; North Carolina Wildlife Resources Commission 2021) across all vegetation types on Grandfather Mountain and none on Roan Mountain Highlands (Figure 6). All seven protected species were detected in the spruce‐fir and six in the ecotone and northern hardwood sites at Grandfather Mountain (Figure 6). Grandfather Mountain had up to an order of magnitude greater bat activity and at least twice as high bat species richness as Roan Mountain Highlands across all forest types (Figure 6).

### 3.4 Vegetation Surveys

Plant species richness (including tree, shrub, and herbaceous species) was highest in the ecotone on both Grandfather Mountain (17 species) and Roan Mountain Highlands (13 species), whereas spruce‐fir forest had the lowest richness on both mountains (6–7 species; Table A6 in Appendix A). On both mountains, at least 38% of tree species in the ecotone were red spruce and/or Fraser fir, with the remainder being northern hardwood trees (Table A6 in Appendix A).

## 4 Discussion

In this study, we surveyed mammal species along the spruce‐fir–northern hardwood forest gradient on two mountains in the Southern Appalachians to better understand their distribution across this environmental gradient and to assess whether the sky‐island specialists currently occupy the spruce‐fir–northern hardwood ecotone. We detected spruce‐fir‐associated small mammals in both spruce‐fir and ecotone habitats, and overall mammal species richness, alpha diversity, and bat activity tended to be higher in the ecotones than in the other forest types. The abundance of small mammals associated with spruce‐fir appeared higher in the spruce‐fir and ecotone forests than in the northern hardwood forest in only one case, although we were not able to estimate abundance for most species–forest type combinations. Collectively, our findings suggest that the ecotone may provide refugium for sky island mammals whose high‐elevation habitat is threatened by climate change.

### 4.1 Mammal Species Richness and Diversity

Mammal species richness and alpha diversity tended to be higher in the spruce‐fir–northern hardwood ecotone on both mountains, highlighting the ecotone's significant conservation value within the biodiversity hotspot of the Southern Appalachian Mountains (Stein et al. 2000; Jenkins et al. 2015). This result is somewhat unsurprising, as ecotones and mid‐elevation montane communities are often known to support disproportionately high biodiversity. Specifically, the positive correlation between plant and animal diversity is well supported in the literature (Qian and Kissling 2010; Castagneyrol and Jactel 2012; Hendel et al. 2024). The ecotones on both mountains were characterized by a high richness of tree and understory species, with over one third of tree species being red spruce or Fraser fir (Table A6 in Appendix A). The spruce‐fir–northern hardwood ecotone thus likely contains critical food resources, cover, microclimates, habitat heterogeneity, and/or structural complexity that may support spruce‐fir‐dependent species and potentially serve as an important refugium as core habitat contracts under climate change.

Species‐specific functional traits can offer insights into how different mammals may utilize the ecotone. For example, species with specialized diets may rely on the ecotone for foraging, particularly where red spruce and Fraser fir remain despite the overall loss of spruce‐fir forests. The Carolina northern flying squirrel and southern red‐backed vole, for instance, depend on hypogeal fungi associated with red spruce (Loeb et al. 2000; Browne and Ferree 2007; Ford et al. 2014; Diggins et al. 2017), while the American red squirrel relies heavily on conifer seeds (Morelli et al. 2025). Other species may use the ecotone because of its structural complexity, such as the Carolina northern flying squirrel requiring mixed red spruce–yellow birch stands for den sites (Ford et al. 2014) and the southern red‐backed vole and woodland jumping mouse relying on moist, complex understories for cover (Browne and Ferree 2007; Linzey 2016).

Importantly, we detected the five spruce‐fir‐associated small mammals (Carolina northern flying squirrel, southern red‐backed vole, woodland jumping mouse, cloudland deer mouse, and American red squirrel) in both the spruce‐fir and ecotone forests. Recent empirical evidence suggests that in spruce‐fir forests of the northeastern United States, American red squirrel populations are not shifting upslope as a result of warmer temperatures from climate change, but rather downslope into the spruce‐fir–northern hardwood ecotone to track habitat needs of red spruce presence and lower precipitation (Morelli et al. 2025). We did not detect all five species in both forest types on both mountains; for example, the woodland jumping mouse was solely detected in Roan Mountain Highland's ecotone. However, this species prefers high‐elevation riparian habitat (Campbell et al. 2010), which only occurred within our ecotone survey site in the Roan Mountains. Nevertheless, our findings collectively suggest that the distribution of spruce‐fir‐associated species extends into the ecotone, rather than being confined solely to the high‐elevation spruce‐fir forest.

### 4.2 Small Mammal Abundance

Rodent abundances varied across forest types and mountains. The patterns of species' abundance were less clear than the results of the other analyses, and we were not able to estimate the abundance of most small mammals due to a low number of captures. The only spruce‐fir‐associated species that tended to follow the expected increase in abundance with elevation was the southern red‐backed vole on Grandfather Mountain. Two non‐exclusive factors that might be driving the observed variation in small mammal abundance (along with mammal richness and diversity) between the two mountains are climate and vegetation. First, Roan Mountain Highlands is taller than Grandfather Mountain (1916 m asl vs. 1812 m asl), and consequently, every site that we surveyed on Roan Mountains was approximately 250 m higher than its equivalent forest type on Grandfather Mountain. For example, the center of the Roan Mountains northern hardwood forest site is at 1591 m, whereas the Grandfather Mountain northern hardwood site is at 1339 m. Low‐elevation species, like the white‐footed mouse, may be rare even in the northern hardwood forest on Roan Mountain Highlands because this area is at the altitudinal limit of the species' temperature tolerance (Linzey 2016). Second, Grandfather Mountain has a higher diversity of plant species than Roan Mountain Highlands in all three forests. For example, the spruce‐fir forest on Roan Mountain Highlands consists of mostly dense Fraser fir trees with minimal understory species, whereas the spruce‐fir forest on Grandfather Mountain consists of a mix of Fraser fir, red spruce, and some yellow birch, and a diverse understory composed of numerous woody and herbaceous plant species. Thus, the spruce‐fir forest on Grandfather Mountain may provide a more complex, productive habitat and warmer microclimate than the one found on Roan Mountain Highlands, hosting larger small mammal populations and more diverse communities (Qian and Kissling 2010; Castagneyrol and Jactel 2012; Hendel et al. 2024).

### 4.3 Flying Squirrel and Bat Activity

Across both mountains, we detected almost all of the Carolina northern flying squirrels in spruce‐fir forests. This finding supports the well‐documented reliance of Carolina northern flying squirrels on spruce‐fir forests (Diggins 2023; Diggins et al. 2017, 2020; Ford et al. 2014). Although this species may select northern hardwood trees such as yellow birch for den sites, mature spruce‐fir forest is preferentially selected as foraging habitat, likely due to the abundance of hypogeal fungi important to the species' diet (Menzel et al. 2004, 2006; Ford et al. 2014; Diggins et al. 2017; Diggins and Ford 2017). Thus, the projected contraction of spruce‐fir sky islands poses a greater threat to Carolina northern flying squirrels than to the other spruce‐fir‐associated species in the Southern Appalachians. Moreover, this species may face increased resource competition, parasite mediation (Weigl et al. 1992), and hybridization from southern flying squirrels, which preferentially select northern hardwood forests (Diggins 2023) and can be common in the ecotone. We suggest that the persistence of Carolina northern flying squirrels in the Southern Appalachians will require continued conservation and restoration of spruce‐fir sky islands, and the conservation of the ecotone may not necessarily benefit this spruce‐fir obligate species.

Bat species diversity and activity differed drastically between the two mountains, with Grandfather Mountain supporting about twice as many species, all the detected species with protected status, and much higher bat activity than Roan Mountain Highlands. The mechanisms underlying the greater number of recorded bat calls on Grandfather Mountain are unclear, but we suspect that habitat availability and temperature are key factors contributing to this variation. Caves and rocky outcrops that may provide suitable hibernacula and roosting habitat for cave bat species (i.e., *Myotis* species and tri‐colored bats *Perimyotis subflavus* ) are relatively common on Grandfather Mountain (Holler Jr et al. 2020), but are lacking on Roan Mountain Highlands. Indeed, only one cave bat species was detected on Roan Mountain Highlands compared to six species on Grandfather Mountain. Another factor is that bat species richness is generally negatively correlated with elevation, as higher elevations are associated with cooler temperatures and lower insect prey availability (McCain 2007; Long 2020; Diggins and Ford 2022). It is possible that Roan Mountain Highlands lies below the lower thermal and energetic limits of smaller bats, such as Myotids (McCain 2007; Long 2020). Previous bat studies in the Southern Appalachian spruce‐fir sky islands are limited, so future research will benefit from long‐term acoustic monitoring to better understand the status and the drivers of high‐elevation bat communities in the region (Graeter et al. 2015; Diggins and Ford 2022).

There are four limitations of this study worth noting. First, we surveyed only one site per vegetation type, all located on the southern aspect of each mountain. Therefore, we lack replication within forest types and across a diversity of topographical features (e.g., sheltered coves), microclimates and aspects. Because of this, patterns in species richness, diversity, abundance, and activity may reflect both differences among forest types and site‐specific characteristics. This caveat also prevented analyses of biotic and abiotic factors that may influence variation in these measures within and across mountains, including historical land use. Future studies should conduct these surveys at multiple sites per forest type, and over several years and mountains to provide a more comprehensive understanding of spatial and temporal patterns of spruce‐fir‐associated species' distributions along the elevational gradient. Second, our surveys were limited to the summer months, so we could not assess potential seasonal variation in mammal species richness, diversity, abundance, and activity across the three forest types. Seasonal patterns are likely less pronounced in this system, however, because many of the focal species reduce activity or hibernate during winter. Additionally, our alpha diversity estimates, based solely on camera‐trapping data, might have been influenced by species behavior, which may have reduced the detectability of some species and biased diversity estimates. Third, we were not able to investigate species' requirements and habitat use within each forest type, and instead focused on species presence and abundance within each forest. Unlike plants and other sessile organisms, mammals are mobile and may shift habitat use frequently, so their presence in ecotones, for example, does not mean that an ecotone is their preferred habitat. Detection of a species only indicates that an individual was trapped or recorded; it does not necessarily reflect long‐term habitat preference or use. Future research should examine niche requirements and habitat use of both high and low‐elevation species, ideally using individually marked animals, to better understand how mobile species use ecotones. Finally, none of our survey techniques targeted shrews (family *Soricidae*), and so this relatively large, important mammal taxon is not included in this study.

Despite these limitations, this study is valuable for mammal conservation in Southern Appalachia. This is the first study of Southern Appalachian mammal communities across the spruce‐fir–northern hardwood forest gradient and the first study in this region to incorporate a diverse mammal survey protocol (i.e., livetrapping, camera trapping, and acoustic recording). Our research provides foundational documentation of mammal species presence, richness, and abundance for the three forest types across this gradient (i.e., spruce‐fir, spruce‐fir–northern hardwood ecotone, and northern hardwood) that can be used as a baseline for future studies seeking to improve understanding of mammal communities in Southern Appalachian spruce‐fir sky islands under climate change.

## 5 Conclusions

This study suggests that in the Southern Appalachian Mountains, mammals associated with sky islands occupy areas outside of the spruce‐fir forest, notably the spruce‐fir–northern hardwood ecotone. Importantly, in many regions, the ecotone is expected to persist longer than the disappearing spruce‐fir forests of the sky islands, underscoring its potential importance as a refugium in this biodiversity hotspot. Although the ecotone may become less climatically suitable as conditions warm, its substantial spruce and fir component likely continues to provide critical structural and resource‐based benefits, such as food and shelter, that are not available in northern hardwood forests alone. These features may allow high‐elevation mammals to persist in the ecotone even as spruce‐fir habitat contracts. We advocate for the conservation of the ecotone forest, in addition to the adjacent spruce‐fir forest, to help support the persistence of high‐elevation mammal communities in the Southern Appalachian Mountains under anthropogenic climate change.

## Author Contributions

**Jenifer A. Mallinoff:** conceptualization (lead), investigation (lead), methodology (lead), writing – original draft (lead), writing – review and editing (lead). **Radmila Petric:** conceptualization (supporting), investigation (supporting), methodology (supporting), writing – review and editing (supporting). **Corinne A. Diggins:** conceptualization (supporting), investigation (supporting), methodology (supporting), writing – review and editing (supporting). **Elizabeth M. Kierepka:** investigation (supporting), writing – review and editing (supporting). **Brian S. Arbogast:** investigation (supporting), writing – review and editing (supporting). **Andrew Jenkins:** investigation (supporting), writing – review and editing (supporting). **Marketa Zimova:** conceptualization (lead), investigation (lead), methodology (lead), writing – original draft (lead), writing – review and editing (lead).

## Conflicts of Interest

The authors declare no conflicts of interest.

## Data Availability

DNA sequences are available in GenBank (Accession Numbers PV487868–PV487919). All data and R scripts are openly available in the Dryad Digital Repository: https://doi.org/10.5061/dryad.sn02v6xjw.

## Appendix A

**TABLE A1:** Mammal survey site characteristics from montane sky islands in the Southern Appalachian Mountains of western North Carolina, USA in summer 2023. Aspect and slope were taken at the center of each live‐trapping grid. Elevation encompasses the range across which all three survey methods (i.e., live‐trapping, camera trapping, acoustic recording) took place within each forest type. See Figure 1 for a schematic of the positioning of these methods within each forest type.

Mountain	Forest	Aspect (°)	Slope (%)	Elevation (m)	Location
Grandfather	Spruce‐fir	175	−21	1564–1635	36.09711, ‐81.82901
	Ecotone	174	−22	1370–1460	36.09060, ‐81.83112
	N. Hardwood	190	−20	1324–1356	36.08582, ‐81.84200
Roan	Spruce‐fir	160	−19	1843–1872	36.10316, ‐82.13133
	Ecotone	145	−14	1625–1659	36.10293, ‐82.10815
	N. Hardwood	180	−17	1547–1617	36.10100, ‐82.10026

**TABLE A2:** Small mammal species abundance estimates from Grandfather Mountain and Roan Mountain Highlands in the Southern Appalachian Mountains of western North Carolina, USA in summer 2023. We estimated population abundances using the Lincoln‐Petersen and Schnabel methods with 95% confidence intervals using the Poisson distribution. The Lincoln‐Petersen abundance estimation method uses the equation *N* = *(C*M)/R* and the Schnabel method uses the equation *N* = *Σ(C * M)/ΣR*, where *C* = number of total captures on each trap occasion, *M* = number of marked individuals on each occasion, and *R* = number of recaptures on each occasion (Schnabel, 1938; Krebs, 1999).

Mountain	Forest	Species	Method	Estimate	95% CI
Grandfather	Spruce‐fir	*Peromyscus maniculatus*	Lincoln‐Petersen	13	6–30
			Schnabel	14	9–25
		*Peromyscus leucopus*	Lincoln‐Petersen	3	1–59
			Schnabel	6	1–118
		*Clethrionomys gapperi*	Lincoln‐Petersen	27	8–152
			Schnabel	16	7–37
	Ecotone	*Peromyscus maniculatus*	Lincoln‐Petersen	22	12–44
			Schnabel	22	14–37
		*Peromyscus leucopus*	Lincoln‐Petersen	4	1–78
			Schnabel	3	1–17
		*Clethrionomys gapperi*	Lincoln‐Petersen	4	1–78
			Schnabel	4	1–23
	N. Hardwood	*Peromyscus maniculatus*	Lincoln‐Petersen	15	8–32
			Schnabel	14	9–26
		*Peromyscus leucopus*	Lincoln‐Petersen	32	14–95
			Schnabel	46	19–135
Roan	Spruce‐fir	*Peromyscus maniculatus*	Lincoln‐Petersen	10	5–24
			Schnabel	9	5–17
	Ecotone	*Peromyscus maniculatus*	Lincoln‐Petersen	16	9–40
			Schnabel	16	9–27
		*Napaeozapus insignis*	Lincoln‐Petersen	2	0–39
			Schnabel	2	1–7
	N. Hardwood	*Peromyscus maniculatus*	Lincoln‐Petersen	80	34–236
			Schnabel	62	34–126

**TABLE A3:** Total counts of individual live‐captured small mammals surveyed at Grandfather Mountain and Roan Mountain Highlands in the Southern Appalachian Mountains of western North Carolina, USA summer 2023. The counts were used to estimate abundance (Table A2).

		*Blarina brevicauda*	*Clethrionomys gapperi*	*Napaeozapus insignis*	*Peromyscus leucopus*	*Peromyscus maniculatus*	*Sorex cinereus*
Grandfather	Spruce‐fir	—	13	—	3	13	—
	Ecotone	—	2	—	3	29	—
	N. Hardwood	1	—	—	24	17	—
Roan	Spruce–fir	—	—	—	—	10	1
	Ecotone	1	2	2	1	15	—
	N. Hardwood	—	—	—	—	33	—

**TABLE A4:** Complete list of the 33 mammal species that were detected at Grandfather Mountain and Roan Mountain Highlands in western North Carolina, USA in summer 2023 using three survey methods (ultrasonic acoustic recorders, remote camera traps, Sherman live‐trap grids).

Family	Common name	Scientific name	Grandfather			Roan			Method of detection
			SF	ECO	NH	SF	ECO	NH	
*Canidae*	Coyote	*Canis latrans*	*	*	*	*	*		Camera trap
*Cervidae*	White‐tailed Deer	*Odocoileus virginianus*	*	*	*	*	*	*	Camera trap
*Cricetidae*	Southern Red‐backed Vole	*Clethrionomys gapperi*	*	*			*		Live‐trap
	White‐footed Mouse	*Peromyscus leucopus*	*	*	*		*		Live‐trap
	Cloudland Deer Mouse	*Peromyscus maniculatus nubiterrae*	*	*	*	*	*	*	Live‐trap
*Didelphidae*	Virginia Opossum	*Didelphis virginiana*					*	*	Camera trap
*Felidae*	Bobcat	*Lynx rufus*	*		*	*		*	Camera trap
*Leporidae*	Cottontail spp.	*Sylvilagus spp*.	*	*		*			Camera trap
*Mephitidae*	Striped Skunk	*Mephitis mephitis*					*		Camera trap
*Molossidae*	Mexican Free‐tailed Bat	*Tadarida brasiliensis*	*	*	*	*	*	*	Acoustic
*Mustelidae*	Long‐tailed Weasel	*Neogale frenata*		*			*		Camera trap
*Procyonidae*	Northern Raccoon	*Procyon lotor*		*	*		*	*	Camera trap
*Sciuridae*	Carolina Northern Flying Squirrel	*Glaucomys sabrinus coloratus*	*			*	*		Acoustic
	Southern Flying Squirrel	*Glaucomys volans*		*	*			*	Acoustic
	Eastern Gray Squirrel	*Sciurus carolinensis*		*	*			*	Camera trap
	Eastern Chipmunk	*Tamias striatus*	*	*	*		*	*	Camera trap
	American Red Squirrel	*Tamiasciurus hudsonicus*	*	*	*	*	*	*	Camera trap
*Soricidae*	Northern Short‐tailed Shrew	*Blarina brevicauda*			*		*		Live‐trap
	Common Shrew	*Sorex cinereus*				*			Live‐trap
*Ursidae*	American Black Bear	*Ursus americanus*	*	*	*			*	Camera trap
*Vespertilionidae*	Rafinesque's Big‐eared Bat	*Corynorhinus rafinesquii*	*	*	*				Acoustic
	Big Brown Bat	*Eptesicus fuscus*	*	*	*	*	*	*	Acoustic
	Eastern Red Bat	*Lasiurus borealis*	*	*			*		Acoustic
	Hoary Bat	*Lasiurus cinereus*	*	*	*	*	*		Acoustic
	Silver‐haired Bat	*Lasionycteris noctivagans*	*	*	*	*	*	*	Acoustic
	Gray Bat	*Myotis grisescens*	*						Acoustic
	Eastern Small‐footed Bat	*Myotis leibii*	*	*	*				Acoustic
	Little Brown Bat	*Myotis lucifugus*	*	*	*		*	*	Acoustic
	Northern Long‐eared Bat	*Myotis septentrionalis*	*	*	*				Acoustic
	Indiana Bat	*Myotis sodalis*	*	*	*				Acoustic
	Evening Bat	*Nycticeius humeralis*	*	*					Acoustic
	Tri‐colored Bat	*Perimyotis subflavus*	*	*	*				Acoustic
*Zapodidae*	Woodland Jumping Mouse	*Napaeozapus insignis*					*		Live‐trap

*Note:* Species presence in each forest type is indicated with an asterisk (*). High‐elevation species are in bold face. For forest type abbreviations, SF stands for spruce‐fir, ECO stands for ecotone, and NH stands for northern hardwood.

**TABLE A5:** Ultrasonic acoustic recorder survey results from summer 2023 for American flying squirrel (*Glaucomys*) species at Grandfather Mountain and Roan Mountain Highlands.

		Grandfather			Roan		
		SF	ECO	NH	SF	ECO	NH
*G. sabrinus coloratus*	Trill	27	—	—	153	—	—
	Chirp	261	—	—	6	4	—
	Upsweep	3	—	—	3	—	—
	Crow	—	—	—	—	—	—
*G. volans*	Trill	—	335	13	—	—	31
	Chirp	—	—	—	—	—	—
	Upsweep	—	—	—	—	—	—
	Crow	—	8	—	—	—	—
*Glaucomys* species	Upsweep	—	—	—	—	1	—

*Note:* Number indicates counts of each call type in each forest type. For forest type abbreviations, SF stands for spruce‐fir, ECO stands for ecotone, and NH stands for northern hardwood.

**TABLE A6:** Vegetation survey summary for survey sites within three forest types on Grandfather Mountain and Roan Mountain Highlands. Species richness is calculated as the total number of plant species detected at each site (including tree, shrub, and herbaceous species). Percentage of tree species are calculated from all tree species present at the survey site.

	Grandfather			Roan		
	Spruce‐fir	Ecotone	Northern hardwood	Spruce‐fir	Ecotone	Northern hardwood
Species richness	7	17	18	6	13	7
% Boreal tree spp. (% fir, % spruce)	94 (83, 11)	44 (0, 44)	0	78 (78, 0)	38 (10, 28)	0
% Hardwood tree spp.	6	56	100	22	62	100
% Boreal sapling spp. (% fir, % spruce)	100 (98, 2)	80 (0, 100)	0	0	0	0
% Hardwood sapling spp.	0	20	100	0	100	100
